# Survival analysis of HER2 receptor negative and HER2 receptor low breast cancer patients at a Philippine Tertiary Government Hospital

**DOI:** 10.3332/ecancer.2025.1840

**Published:** 2025-02-06

**Authors:** Carl Arenos, Steven Lim, Andreu Lominoque, Isabela Reveldez, Angeli Sison-Dimaano, Nehar Pagandaman, Vincent Tatoy, Timothy Uy, Michael San Juan

**Affiliations:** Division of Medical Oncology, Department of Medicine, University of the Philippines-Philippine General Hospital (UP-PGH), Manila 1008, Philippines; a https://orcid.org/0009-0001-6070-6924

**Keywords:** breast cancer, Her2-low, prognosis

## Abstract

**Background and objectives:**

Human epidermal receptor 2 (HER2)-low breast cancer, characterised by specific immunohistochemistry (IHC) scores (+1 or +2) or negative fluorescence in-situ hybridiszation (FISH), has unique biological traits, therapeutic outcomes and prognosis. Recent studies highlight the effectiveness of Trastuzumab-Deruxtecan (T-Dxd) for HER2-low patients. This study seeks to deepen understanding of HER2 negative and low patients for tailored treatment by determining the 3-year disease-free survival (DFS) rates and prognostic variables of different subsets of HER2 negative (HER2 0) and HER2-low breast cancer patients (HER2 +1, HER2 +2 and HER2 FISH negative).

**Methodology:**

We analysed the records of 138 patients with non-metastatic breast cancer, exhibiting HER2 IHC 0, +1 or +2/FISH negative. Data on 3-year DFS in months, age, stage (early stage versus locally advanced) and menopausal status were collected. Kaplan–Meier survival curves were used to plot 3-year DFS, while Cox Proportional Analysis assessed the prognostic significance of age and menopausal status in the HER2-low population.

**Results:**

Three-year DFS for HER2 0 was 84.4%, HER2 +1 was 81.8% and HER2 +2/FISH negative was 52.9%, displaying statistically significant differences (*p* = 0.00012). Subgroup analyses revealed consistently worse DFS for HER2 +2/FISH negative patients in both early and advanced stages (*p* = 0.0042, 0.0057). Cox proportional analysis showed a recurrence hazard ratio of 4.0–4.5 for HER2 +2/FISH negative patients. Among prognostic factors, post-menopausal status correlated with a decreased risk of recurrence (HR 0.4387), signifying a 56.13% lower recurrence risk compared to pre-menopausal patients (*p* = 0.00723). On the other hand, patient age was not correlated with a reduced risk of recurrence (HR 0.97; *p* = 0.0544).

**Conclusion:**

This study reveals a worse 3-year DFS in HER2 +2/FISH negative patients across both early and advanced disease stages. The findings highlight the prognostic importance of HER2-low status and may guide future therapeutic strategies, including the use of targeted therapies like T-Dxd in non-metastatic patients.

## Introduction

### Background and review of related literature

Cancer is one of the four epidemic non-communicable diseases in our country, affecting 189 of every 100,000 Filipinos with four patients dying every hour due to the condition [1]. According to the latest Global Cancer Observatory data, breast cancer is the most prevalent cancer in the world amounting to 8.1 million women worldwide diagnosed with the condition in the past 5 years [2]. In the Philippines, breast cancer comprises up to 17.5% of all newly diagnosed cases and is the leading cause of cancer-related death in the country [2]. A considerable factor in the high rates of breast cancer incidence is the lack of awareness/education about the disease [3].

One of the adjuncts in the treatment and prognostication of breast cancer patients is the determination of hormone status, such as estrogen (ER) and progesterone receptors (PR), and human epidermal receptor 2 (HER2) receptors. These are determined using immunohistochemistry (IHC) or in-situ hybridisation (ISH). IHC is used to characterise cellular characteristics using certain markers that detect intracellular proteins and cell surfaces. IHCs can be used to guide treatment and prognostic decisions by determining tumour subtypes, cell origin and distinguishing metastatic from primary tumour.

The most common IHC markers used in breast cancer are ER, PR, Ki-67 antigen and HER2/Neu expression. Breast cancer tissues are considered ER or PR positive if at least 1% of the examined cells are stained. Ki-67 antigen is expressed by cells in the G1, S, G2 and M phases of the cell cycle and is used as an index of proliferation of neoplastic cells. It is expressed as a percentage of positive cells in a tissue sample.

HER2 receptor is a tyrosine kinase that has effects on cell proliferation and survival. In breast cancer patients, HER2 overexpression is one of the main catalysts for cancer cell progression in a subset of breast cancer patients [4–8]. Patients who test positive for HER2 receptors can avail of treatment of HER2 receptor antagonists such as Trastuzumab, Pertuzumab and Lapatinib. HER2 expression ranges from HER2 positive (with a score of +3) to HER2 negative (score of 0).

The American Society of Clinical Oncology/College of American Pathologists defines the HER2 spectrum as:

HER2 score 3+ is defined as complete membrane staining, intense and >10% of tumour cells. HER2 score 2+ is defined as weak-moderate complete membrane staining in >10% of tumour cells or membrane staining is intense but in ≤ 10% of tumour cells. HER2 score 1+ qualifies as incomplete membrane staining that is faint or barely perceptible and >10% of tumour cells. HER2 ultra-low is outlined as incomplete membrane staining and faint/ barely perceptible in <10% of tumour cells. HER2 negative refers to samples that have no staining results upon analysis.

Currently, an evolving trend in cancer is the clinical implication of HER2-low breast cancer. It is defined as breast cancer subtypes with HER2 IHC score of 1+ or 2+ with a negative ISH result [4–8]. Approximately more than half of breast cancers may be classified under HER2 low subtype. HER2-low patients were noted to have distinct biological characteristics and show different outcomes in terms of therapy and prognosis. Current literature demonstrates conflicting results over the years, which shows that more studies are needed to shed more light on this matter.

Survival studies have been done comparing HER2 negative and HER2 low patients. It was seen that HER2 negative and HER2 low patients have similar prognosis regardless of hormone receptor status [9, 12] but it was noted that HR-positive tumours with HER2 low status have better disease-free survival (DFS) rates than HR-positive/ HER2 negative [9]. While further analysis of survival rates between HER2 negative and HER2 low patients show that patients with the latter have a longer DFS and overall survival (OS) compared to the former [10, 11]. Certain applications regarding HER2 low have been applied to patients. In a study involving HER2 low breast cancer patients, there was no prognostic value [13]

Recent studies show the effectiveness of certain medications for patients diagnosed with HER2-low cancers. In particular, the anti-HER2 antibody-drug conjugate Trastuzumab-Deruxtecan (T-Dxd) has been proven to provide survival benefits for this subset of patients [14, 15].

### Implications and importance of the study

This study highlights the importance of HER2 negative and HER2 low patients in terms of prognostication and determines which subset of patients will benefit from emerging regimens targeted at HER2 low patients.

### Research question

Is there a difference in the DFS between non-metastatic breast cancer patients diagnosed with HER2 negative status and those with HER2 low status?

### Objectives

#### General objectives

Determine the 3-year DFS of different subsets of HER2 negative (HER2 0) and HER2 low breast cancer patients (HER2 +1, HER2 +2 and HER2 fluorescence *in-situ* hybridisation (FISH) negative).

#### Specific objectives

1. Analyse the association between triple-negative breast cancer (TNBC) with HER2 negative and HER2 low patients with respect to DFS.
2. Analyse the DFS of HER2 negative and HER2-low patients on early-stage breast cancer patients.
3. Analyse the DFS of HER2 negative and HER2-low patients on locally advanced breast cancer patients.
4. Determine the relation of other variables, such as age and menopausal status in the prognosis of HER2 low patients.

## Methodology

### Study design

This is a retrospective cohort study on HER2 negative and HER2 low patients diagnosed in 2020 or earlier.

### Study setting

The Medical Oncology clinic of the Philippine General Hospital Cancer Institute was the study setting. Patient charts (physical and electronic) were retrieved with the assistance of the Cancer Institute Medical Records Division. The Medical Records Division of the Cancer Institute served as the provider of patient records in this study because they are responsible stewards of patient records within the institute.

### Study population

A total of 138 patient records were included in the analysis [16]. Subjects included in the study are: 1) Patients aged more than 18 years old; 2) Breast cancer patients diagnosed from 2017 or earlier; 3) Resected non-metastatic breast cancer patients tumour, node and metastasis (TNM stage from I to IIIC) 4). IHC results bearing HER2 status 0, +1 or +2/equivocal with a FISH result of ‘HER2 not detected.’

**Figure 1.**
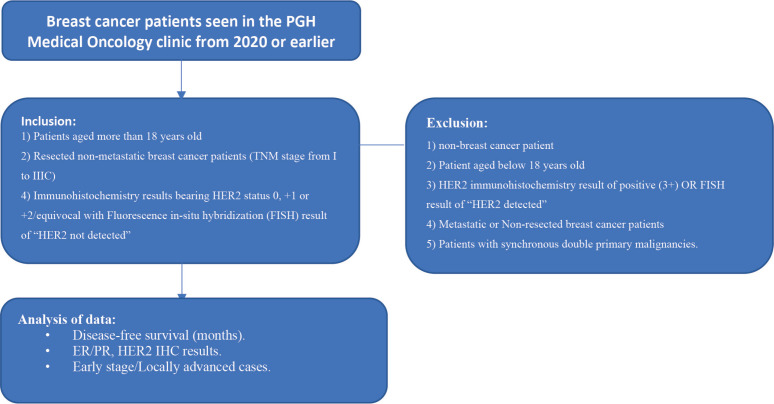
Study flow procedure.

Patients were excluded from the study if they present with any of the following: 1) non-breast cancer patients; 2) Patient aged below 18 years old; 3) HER2 IHC result of positive (3+) OR FISH result of ‘HER2 detected’; 4) Metastatic or non-resected breast cancer patients; 5) Patients with synchronous double primary malignancies (Figure 1).

### Data collection

The study was conducted from 01 May to 18 August 2023. Patient charts of patients diagnosed with breast cancer from the year 2020 or earlier were included in the study. The earliest patient enrolled was diagnosed with breast cancer on October 2002. All investigators of the study collected the predetermined data using electronic medical records and/or physical medical records of the patients. The following data were obtained from the patient’s record: Name, age, cancer type, year of diagnosis, TNM stage, intrinsic subtype (Luminal status, HER2 and Basal-like), neo/adjuvant chemotherapy history, menopausal status and record of disease progression (radiographic or histopathologic) or mortality. The TNM staging will be based on the American Joint Committee on Cancer Staging Manual 7th Edition [17].

The HER2 status was based according to the American Society of Clinical Oncology/College of American Pathologists:

HER2 score +3 is defined as complete membrane staining, intense and >10% or tumour cells. HER2 score +2 is defined as weak-moderate complete membrane staining in >10% of tumour cells or membrane staining is intense but in ≤ 10% of tumour cells. HER2 score 1+ qualifies as incomplete membrane staining that is faint or barely perceptible and >10% of tumour cells. HER2 negative refers to samples that have no staining results upon analysis. Those samples that fall under Her2 +1/+2/FISH negative will be categorised under HER2-low status.

The intrinsic subtype definition was based on the Clinicopathologic Surrogate definition by the European Society of Medical Oncology Clinical Practice Guidelines for Early Breast Cancer [18].

Luminal A intrinsic subtype will correspond to patients with clinicopathologic surrogate definition fulfilling the following:

Luminal A-like: ER-positive, HER2-negative, Ki67 low, PR high (cut-off value of >20%) and low-risk molecular signature (if available).

Luminal B intrinsic subtype will correspond to patients with clinicopathologic surrogate definition fulfilling the following:

Luminal B-like (HER2-negative): ER-positive, HER2-negative, Ki67 high OR PR-low (cut-off value of <20%) and high-risk molecular signature (if available).

Luminal B-like (HER2-positive): ER-positive, HER2-positive, Any Ki67 or any PR status.

HER2 intrinsic subtype refers to patients with HER2-positive and ER/PR-negative results.

Basal-like intrinsic subtype will refer to patients with triple-negative profile (ER/ PR and HER2--negative results).

Menopausal status is defined in this study are individuals who fulfill any of the following criteria: 1) more than 60 years of age; 2) underwent bilateral oophorectomy/irradiation; or 3) patients who are amenorrheic for more than 1 year with a hormonal status within the menopausal range upon surveillance testing (follicle stimulating hormone, luteinising hormone and estradiol serum levels).

The data were extracted from physical charts and electronic medical records from the PGH Medical Records Division. All patient data will be kept in confidentiality with their corresponding alphanumeric patient codes. The electronic data will be recorded and stored using Microsoft Excel.

### Data analysis

Descriptive statistics for summarising the categorical data, correlation analysis and Kaplan–Meier curve were used for statistical analysis. The confidence interval used is 95% (*p*-value cut-off of 0.05). Pearson’s chi-squared test was used to establish if the HER2-low and HER2-negative subgroups are comparable in terms of treatment allocation, confirming the population’s homogeneity for subsequent survival and prognostic analyses. DFS is defined as the length of time a patient or cohort survives with no evidence of primary or metastatic disease. The time from diagnosis to the recurrence of tumour was detected pathologically (via biopsy) or radiographically using imaging studies (CT scan, PET-CT and bone scan). OS pertains to the length of time from the date of diagnosis that patients diagnosed with cancer which are still alive [19, 20]. The data collected was analysed using the Rstudio package. Kaplan–Meier survival curves were used to plot the DFS data and Cox proportional analysis was used to account for the other factors related to the patients such as age and menopausal status.

## Ethical considerations

### Ethics approvals

The study is approved by the University of the Philippines Manila Research Ethics Board (UPMREB) with the corresponding UPMREB CODE: 2023-0189-01.

### Anonymity and privacy/data protection plan

All the data that were retrieved from the medical records of the included patients are kept with utmost anonymity and privacy. The data gathered are stored in a computerised database accessible only to the investigators.

All information collected from the medical records, including their name, age and contact numbers, are kept confidential and accordingly assigned to unique alphanumerical case identifiers upon encoding into the dataset (de-identification and use of coded data).

Unique alphanumerical case identifiers are assigned for each patient and are used in data encoding and analysis. Only the investigators have access to the control numbers assigned for each patient.

All electronic data were stored and secured by the primary investigator in a password-protected and encrypted hard drive and will also be stored until the end of the manuscript writing. In case of a breach of privacy/confidentiality despite de-identification and use of coded information, the primary investigator will review to confirm if a breach of confidentiality was indeed done and accordingly seek legal consultation for the following steps. The PGH Data Privacy Officer will also be accordingly informed.

### Possible benefits, risks and hazards

Since there is no direct interaction with the study population, there are no expected risks to the study population.

The results of this study could benefit breast cancer patients who present with HER2-low IHC results. It will guide future patients with additional possible therapeutic regimens that would fit their breast cancer subtype.

Preliminary results will be relayed to the primary physicians and concerned parties.

### Recruitment process and consent to participate

Patient records using physical charts and electronic medical records were used for this study, no actual patient participation was required.

### Compensation

(not applicable).

### Research funding, conflicts of interest and disclosures

All research-related expenses were covered by the primary investigator. The principal investigator and co-investigators have no disclosures or conflicts of interest related to the study.

### Data dissemination plan

After completion of the study, efforts will be made to have the study published in a local or an international journal. It will also be submitted for presentation in scientific meetings of both local and international societies, such as the Philippine Society of Medical Oncology, American Society of Clinical Oncology and the European Society for Medical Oncology Asia. The results of the study will not, in any form, cause stigma or any negative impact the well-being of the breast cancer patient community.

### Good clinical practice certification

The investigators ensured that their good clinical practice certification is updated prior to and during the implementation of the study.

### Ethics review board and approval

In compliance with the university and hospital regulations, this study protocol was submitted to the University of the Philippines Manila Research Ethics Board (UPMREB) for review and approval.

A waiver of informed consent was requested from the UPMREB panel since (1) the investigators only used hospital medical records (electronic and physical); (2) there are no risks associated with the study since there is no active involvement of human participants (National Ethical Guidelines for Health and Health-Related Research 2017 guidelines provision 16.2.1-4,17.1;4). The investigators ensured that the highest standards of confidentiality and anonymity of patient data will be strictly implemented in accordance with the National Ethical Guidelines for Health and Health-Related Research 2017.

## Results

The results were tabulated and analysed using Microsoft Excel and RStudio statistical software (see Appendix). This study determined if there is a statistical difference in the DFS of HER2 0 versus HER2 low (+1 and +2/FISH negative) subset with further subgroup analysis between Her +1 and HER2 +2/FISH negative. Subgroup analysis on early stage and locally advanced breast cancer, luminal and TNBC were also performed. Cox proportional analysis was used to account for the patient-related factors such as age and menopausal status.

A comprehensive review of 138 patient records was conducted at the Medical Oncology clinic. The study population's mean age was determined to be 55 years, with a standard deviation of 10.4, encompassing an age range from 26 to 74 years of age. Within the cohort, 96 patients were identified with early-stage breast cancer, while the remaining 42 were classified as having locally advanced disease (Table 1). In the early stage group, 83% of patients underwent adjuvant cytotoxic chemotherapy while 96% of patients eligible for adjuvant hormonal therapy were given. In the locally advanced breast cancer cohort, 57% of patients underwent neoadjuvant cytotoxic chemotherapy while the rest (42.86%) underwent adjuvant cytotoxic chemotherapy. Almost all patients (except for 1 patient) in the luminal group received adjuvant hormonal therapy. None of the patients in the study were given adjuvant Cyclin-dependent kinase (CDK) 4/6 inhibitors because access to these drugs were limited (logistically and financially) in our institution.

The distribution of HER2 status among the study population was as follows: 68% were HER2 0 by IHC, and 16% each were classified as HER2 +1 and HER2 +2/FISH negative. The Pearson’s chi squared test analysis was performed which indicated that the study population exhibited homogeneity in terms of HER2 status and treatment allocation. The test revealed no statistically significant association between HER2 status and the administration of neoadjuvant or adjuvant therapy, with p-values of 0.531 and 0.2194, respectively. This lack of association suggests that the HER2 expression levels did not influence the choice of therapy, and therefore, patients in this cohort were not selectively treated with either neoadjuvant or adjuvant therapy based on their HER2 status. These findings support the homogeneity of the study population concerning HER2 status and treatment strategy, allowing us to proceed with the analysis of DFS and prognostic factors.

A detailed breakdown of molecular subtypes revealed that 84% of the study group consisted of Luminal subtypes, whereas the remaining 16% were characterised as basal type or triple negative. It must be noted that the Luminal subtype population could not be further subdivided into Luminal A and Luminal B classifications, due to the absence of Ki-67 IHC data within the majority of the patient records. Menopausal status data was also recorded with the majority of the patients (*n* = 79) were post-menopause while the rest of the sample population was pre-menopausal. The results of statistical analysis generated by the RStudio software were based on a 95% confidence interval.

**Figure 2.**
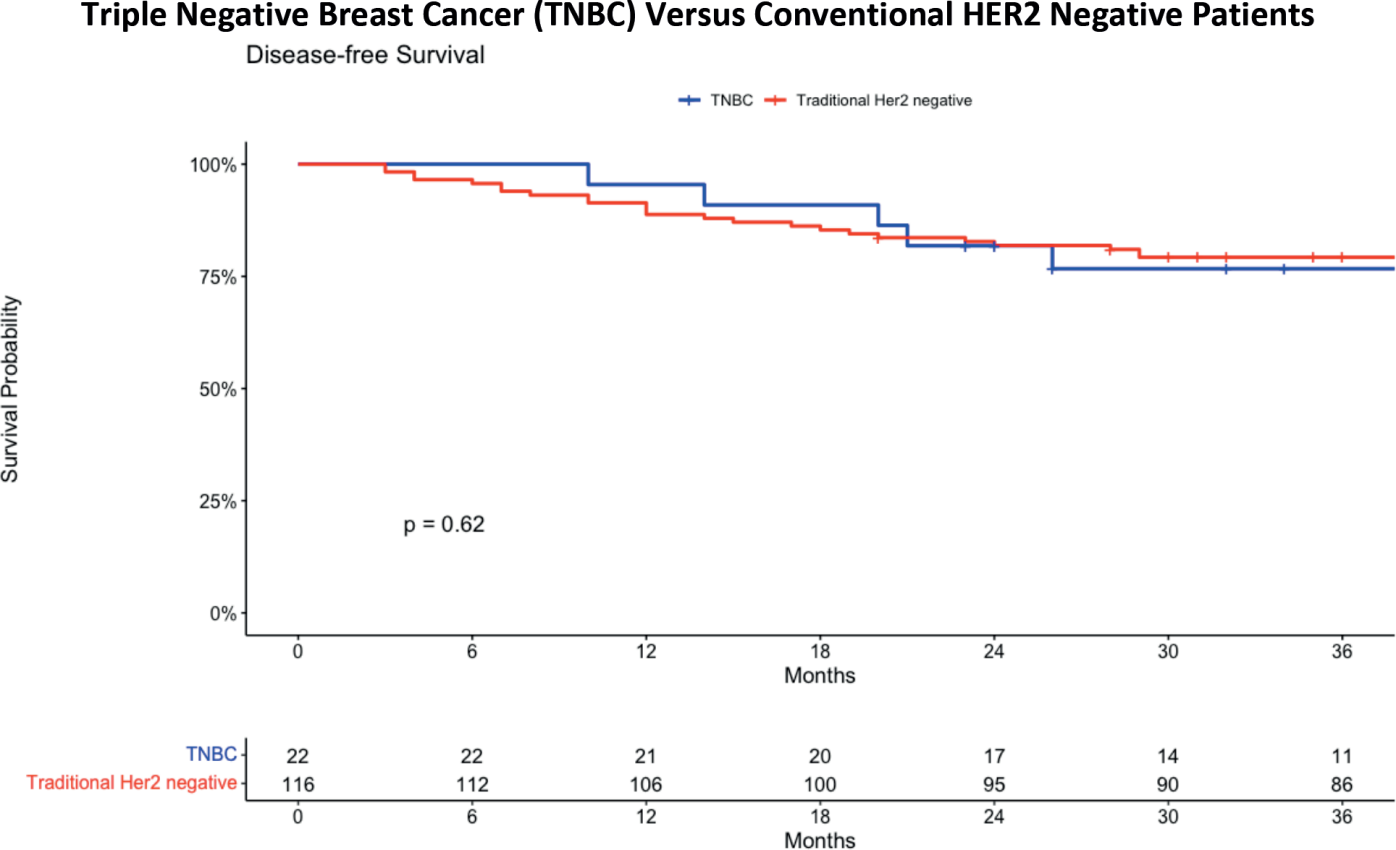
DFS comparing TNBC and traditional HER2 negative patients.

Figure 2 shows an overview of the 3-year DFS curves for patients with TNBC and conventional HER2-negative patients. The latter refers to the original group identified with a HER2 negative status, encompassing HER2 0, HER2 +1 and HER2 +2 as determined by IHC results. This shows an estimate of the general survival curve of patients in the cohort group. For the TNBC group, the 36-month DFS was 76.7%, with a standard error of 0.0916 and a confidence interval ranging from 60.7% to 96.9%. In contrast, the conventional HER2 negative group exhibited a 36-month DFS of 79.2%, with a standard error of 0.0377 and a confidence interval from 72.2% to 87.0%. When comparing the survival curves of these two groups, no statistically significant difference is observed, as evidenced by a *p*-value of 0.62.

**Figure 3.**
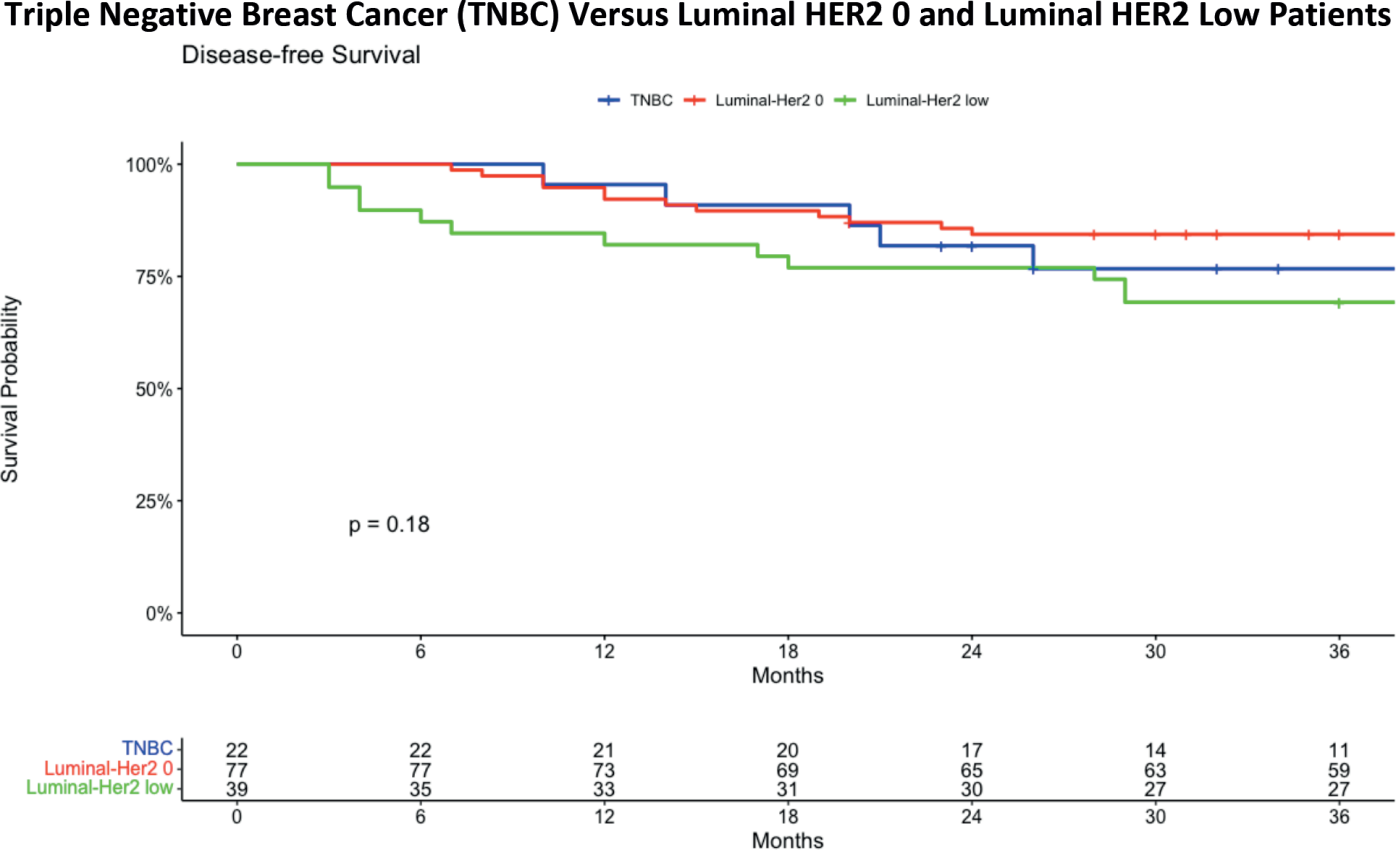
DFS comparing TNBC with luminal HER2 0 and luminal HER2-low patients.

Figure 3 presents the Kaplan–Meier survival curve in which the conventional Luminal HER2 negative group is further subdivided into Luminal-HER2 0 and Luminal HER2-low categories. In the TNBC group, the 36-month DFS was 76.7%, with a standard error of 0.0916 and a confidence interval ranging from 60.7% to 96.9%, showing a gradual decrease in survival over the 36-month period. The HER2 0 group exhibited a more stable survival rate, with a 36-month DFS of 84.4%, a standard error of 0.0414 and a confidence interval from 76.6% to 92.9%. In contrast, the HER2 low group experienced a more pronounced decrease in survival, with a 36-month DFS of 69.2%, a standard error of 0.0739 and a confidence interval from 56.2% to 85.3%. When analysing the 3-year DFS data for patients with either HER2 0 or HER2 low results, no statistically significant difference is found at a *p*-value of 0.18.

**Figure 4.**
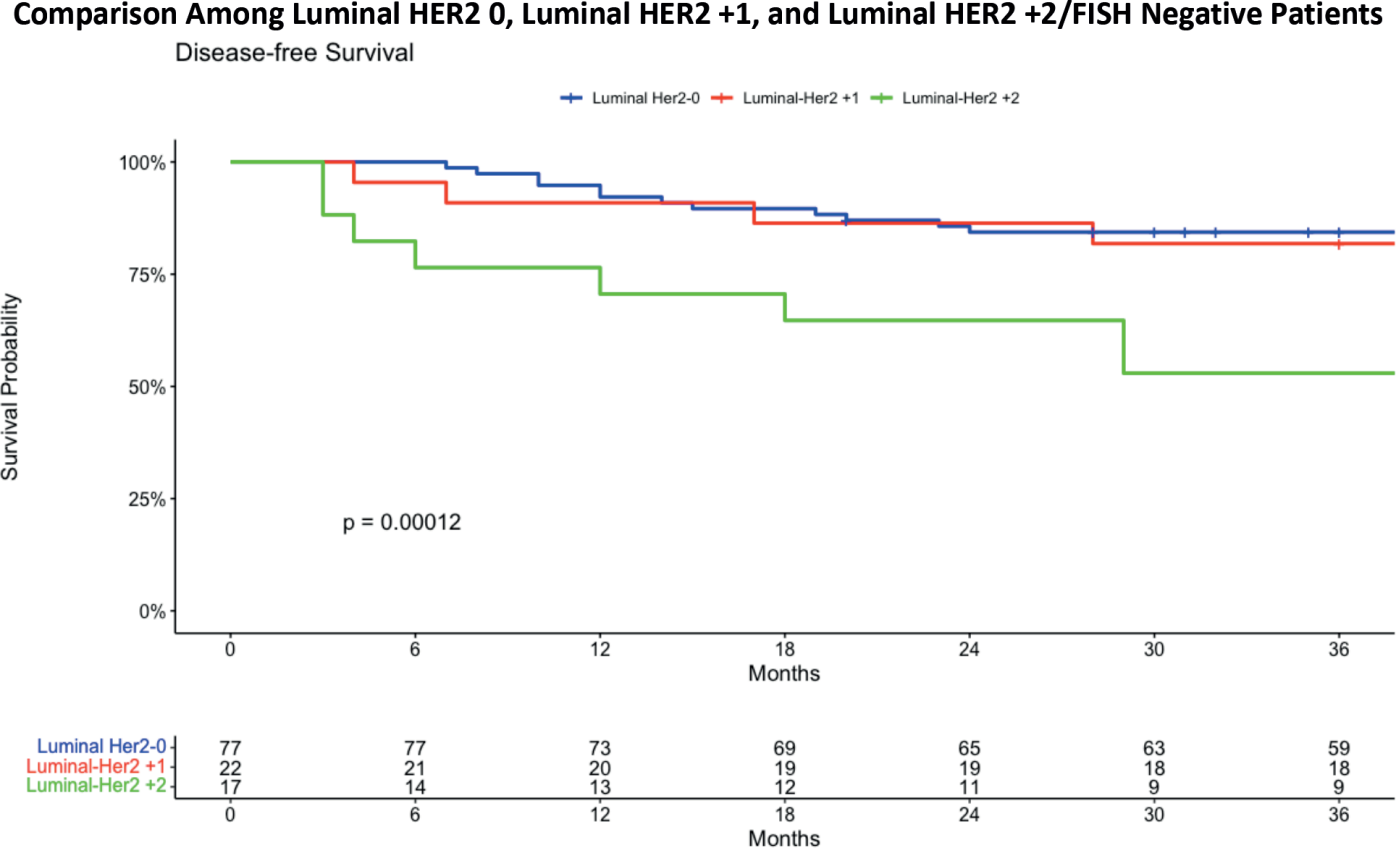
DFS comparing luminal HER2 0 with luminal HER2 +1 and luminal HER2 +2.

The study group has further broken down the Luminal HER2-low patients into Luminal HER2 +1 and Luminal HER2 +2/ FISH negative (Figure 4). The 36-month DFS analysis for three Luminal HER2 subgroups reveals distinct outcomes. The Luminal HER2 0 group exhibited a DFS of 84.4%, with a confidence interval ranging from 76.6% to 92.9%. The Luminal HER2 +1 group showed a slightly lower DFS at 81.8%, with a confidence interval from 67.2% to 99.6%. Most notably, the Luminal HER2 +2/FISH negative group demonstrated a significantly lower DFS at 52.9%, with a confidence interval from 33.8% to 82.9%. These findings underscore the varying survival outcomes among these subgroups, highlighting the lower survival rate in the HER2 +2/FISH negative group over a 36-month period. The statistical evaluation revealed a statistically significant difference in survival outcomes among the groups, with a *p*-value of 0.00012. This finding suggests a worse DFS in patients with a Luminal HER2 +2/FISH negative status.

**Figure 5.**
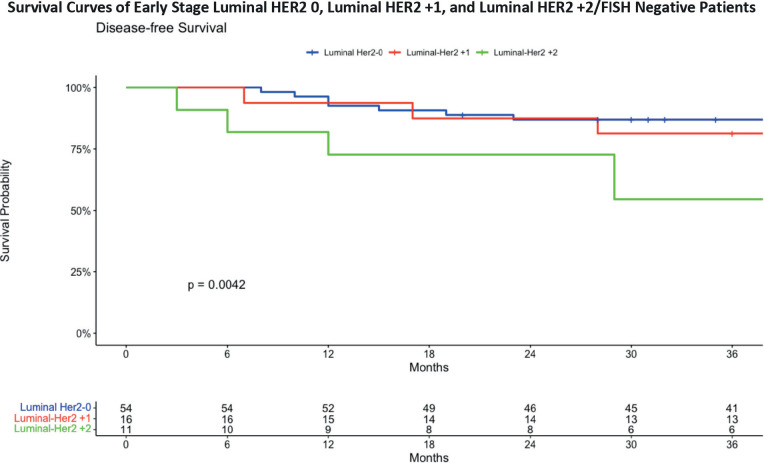
DFS in early stage breast cancer patients with HER2 0, HER2 +1 and HER2 +2/FISH negative status.

The 3-year DFS analysis for early-stage (Figure 5) Luminal HER2 subgroups provides insights into the survival outcomes for these patients. For the Luminal HER2 0 group, the DFS was 87.0%, with a confidence interval ranging from 78.5% to 96.5%. In the Luminal HER2 +1 group, the DFS was slightly lower at 81.2%, with a confidence interval from 64.2% to 100%. The Luminal HER2 +2/FISH negative group demonstrated the lowest DFS at 54.5%, with a confidence interval from 31.8% to 93.6%. These results emphasise the differences in survival outcomes among the early stage Luminal HER2 subgroups, with the HER2 +2/FISH negative group showing a notably lower survival rate over the 36-month period. The data shows a statistically significant difference in terms of DFS in early stage breast cancer patients with Luminal Her 0, HER2 +1 and HER2 +2/FISH negative status. The Kaplan–Meier curve for Luminal HER2 +2 patients shows a significant downtrend compared to other groups.

**Figure 6.**
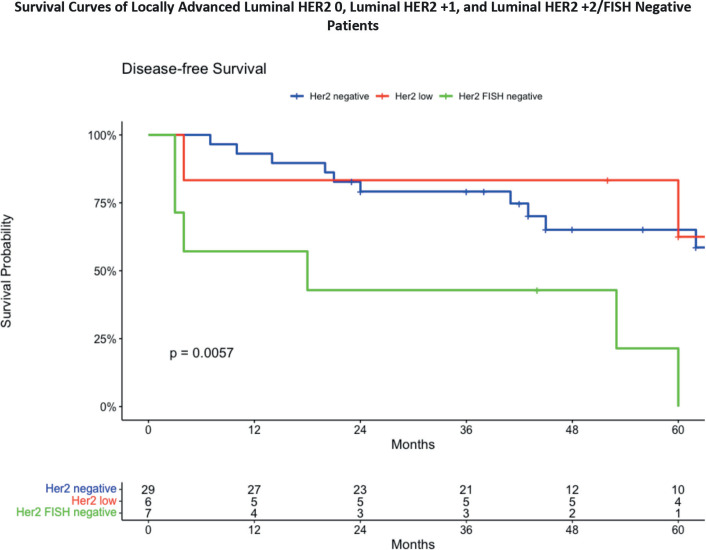
DFS in locally advanced breast cancer patients.

The 3-year DFS analysis for locally advanced (Figure 6) Luminal HER2 subgroups reveals distinct survival outcomes for these patients. In the Luminal HER2 0 group, the DFS was 78.3%, with a confidence interval ranging from 63.1% to 97.1%. For the Luminal HER2 +1 group, the DFS was 83.3%, with a confidence interval from 58.3% to 100%. The Luminal HER2 +2/FISH negative group exhibited the lowest DFS at 50.0%, with a confidence interval from 22.5% to 100%.

These results indicate that locally advanced Luminal HER2 0 and HER2 +1 patients have relatively similar survival outcomes, while the HER2 +2/FISH negative group demonstrates a significantly lower survival rate over the 36-month period. Figure 6 presents a notable statistically significant downward trend in the DFS of patients in patients with HER2 +2/FISH negative patients compared to HER2 negative and HER2 low patients (*p* = 0.0057).

## Cox proportion analysis

To understand the complex relation of factors that influence DFS outcomes, our study employed a Cox Proportional Hazards analysis in the HER2-low groups to investigate the role of age and menopausal status. These variables were selected based on their potential biological and clinical relevance, as well as their prominence in existing literature.

The Cox Proportional Hazards model while controlling for the effect of age (Table 3) revealed distinct effects of HER2 status and age on DFS. Having a HER2 +1 subtype was associated with a negligible decrease in hazard risk of the event compared to the HER2 negative reference group, but this was not statistically significant (*p*-value = 0.9913). Conversely, the HER2 +2/FISH negative showed a substantial increase in hazard, with a risk roughly 350.3% higher than the reference group, and this effect was highly significant (*p*-value = 3.37e-05). Age also appeared to influence the hazard, with a 2.98% lower risk for every 1-year increase, though the result was not statistically significant (*p*-value = 0.0544). The model's concordance of 0.665 indicates good discrimination, and the highly significant global tests (*p*-values ranging from 7e-05 to 9e-04) provide strong evidence that at least one predictor has a significant effect. Overall, the analysis underscores that having a HER2 +2/FISH negative status is a significant predictor of the hazard in this sample with a hazard ratio (HR) of 4.5, while the effects of HER2 +1 status and age were not conclusively demonstrated.

In the conducted Cox proportional hazards analysis (Table 3) while controlling for the effect of menopause, three variables were examined: HER2 +1 status (Group B), HER2 +2 status (Group C) and menopausal state (Menopause), with HER2 negative status serving as the reference category. The analysis revealed that HER2 +1 status was not statistically significant in influencing the hazard, with a hazard ratio (HR) of 0.8552 (*p*-value = 0.70482). In contrast, HER2 +2/FISH negative subtype was associated with a significant increase in hazard, with an HR of 4.0145 (*p*-value = 6.73e-05), with a 300% increase in the risk for recurrence. The menopausal state was also found to be a significant predictor, associated with a decreased hazard, with an HR of 0.4387 (*p*-value = 0.00723). The hazard ratio (HR) of the menopausal state indicates that being in menopause is associated with a decreased risk of recurrence. In other words, the risk is roughly 56.13% lower for post-menopausal individuals compared to pre-menopausal individuals.

The model's concordance of 0.675 and significant global tests (*p*-values ranging from 1e-05 to 1e-04) indicate a robust fit. Overall, the findings suggest that HER2 +2 status and menopausal state are significant predictors in the model, while HER2 +1 status does not appear to have a significant effect.

## Discussion

The comprehensive analysis of 3-year DFS in various patient cohorts revealed mixed findings. While there was no statistically significant difference between TNBC and the conventional luminal HER2-negative patients. Further analysis subdividing the conventional HER2 negative group into HER2 0 and HER2 low patients also shows no significant difference in the 3-year survival analysis. A more detailed breakdown of the Luminal HER2-low group into Luminal HER2 +1 and Luminal HER2 +2/FISH negative revealed a significant difference in survival outcomes (*p*-value = 0.00012), with worse 3-year DFS in the Luminal HER2 +2/FISH negative group. The luminal cohort was divided into early stage and locally advanced breast cancer patients. A significant difference in DFS was observed among early-stage breast cancer patients with Luminal HER2 0, HER2 +1 and HER2 +2/FISH negative status. The Luminal HER2 +2/FISH negative group, in particular, showed a significant downtrend in DFS probability for both early stage and locally advanced cancer patients.

In summary, the analysis highlights the complexity of HER2 status in predicting DFS, with significant variations observed in specific subgroups. The findings underscore the importance of nuanced categorisation and understanding of HER2 status in breast cancer prognosis and treatment planning. The statistically significant differences in some categories emphasise the need for further investigation and potential consideration in clinical decision-making particularly in the HER2-low population.

In the conducted Cox proportional analyses, the HER2 +2/FISH negative group presented with a statistically significant increase the risk for recurrence (HR 4.0–4.5) amounting to an increased risk of 300%–350% when compared to HER2 0 and HER2 +1 group. Adjusting for the effect of age, it was seen that age appeared to influence the risk of recurrence, with a 2.98% lower risk for every 1-year increase in age, although this finding is not statistically significant (*p*-value 0.0544). Cox proportion analysis for menopause showed that there is a 56.13% lower risk for recurrence in post-menopausal patients compared to pre-menopausal patients.

In terms of prognosis, large-scale studies done overseas presented evidence that HER2 low breast cancer patients present with different tumour characteristics depending on the hormone receptor positive status. HER2-low breast cancer patients with the HR-positive subtype showed fewer T4 tumours, higher histologic profile and negative lymphatic invasion in contrast to HER2-low TNBC patients who have higher lymph node ratios and presentation of positive lymphatic invasion. What is notable in this study is that HER2-low breast cancer patients show significantly better breast cancer-specific survival rates compared to HER2 0 breast cancer patients irrespective of HR status [21]. Other studies show no significant difference in the survival rates between HER2 0 and HER2 low patients. A recent study done in China by Zhu *et al* [22] also showed mixed results. Out of 969 cases, 777 were hormone receptor positive and 192 were hormone receptor negative. In the hormone receptor positive group, HER2 low expressors showed no significant difference in the DFS and OS when compared to HER2 0 patients despite the former having a younger study population, higher pre-menopausal patients and more lymph node involvement.

In the hormone receptor-negative group, there was a better DFS in the HER2-low expressors when compared to the HER2 0 group but the OS benefit was not proven here [22]. Two meta-analyses comparing HER2 and HER2 low patients was done for early stage breast cancer patients. This study compiled 600 thousand cases and showed that having a HER2-low status is associated with an improved DFS and OS regardless of hormone receptor status [23–25]. In terms of the statistically significant worse DFS in HER2 +2/FISH negative patients, there are currently no studies that observed the same trend seen in this study as well as the absence of studies that divided the analysis of HER2 low patients into Hr2 +1 and HER2 +2/FISH negative. Perhaps this could be an avenue for further research in the future.

The cox proportional analysis for age showed that the risk for breast cancer recurrence is decreases by approximately 3% for each increase in the age of the patient. An analysis of the Surveillance, Epidemiology and End Results database from 2004 to 2008 showed that younger patients aged less than 40 years old showed worse OS and breast cancer-specific survival. What is noteworthy in this study is that further analysis showed an age of 60 and above was a significant independent predictor for poor prognosis [26]. A study with a 10-year follow-up during 1961–1991 investigated the link between age of diagnosis and breast-cancer-specific mortality. Analysis done in this study showed that patients aged less than 40 and older than 80 had a statistically significant 1-year mortality rate when compared to women aged 40–49 years old. Similar trends could not be replicated in our study because the oldest patient in the cohort is only 74 years of age [27]. A similar trend was seen in a nationwide cohort study done in Denmark which showed that the cumulative incidence of late recurrence was higher in younger patients compared to older patients. With adjusted hazards of recurrence for women who were diagnosed before 40 years of age [28].

In terms of menopausal status, the results seen in this study were consistent with a few studies. In a retrospective study done in 2022, post-menopausal patients were seen to have better DFS in the Kaplan-Meier curve in contrast to pre-menopausal patients with HER2 enriched, Luminal/HER2 type,

Luminal A and triple-negative subtype, although this trend was only statistically significant for the triple-negative subtype (*p* = 0.01) [29]. However, another study done by Lao *et al* [30] on women with stage I–III breast cancer patients aged 44–55 years old demonstrated that menopausal patients with ER+ and/or PR + subtypes have a higher risk for metastatic relapse at an HR of 1.38. Although this was not proven to be true as well for women with ER and PR-negative disease [30]. A commentary done on this study that pointed out certain limitations like the age range of patients within the study and the absence of their treatment details of the individual patients. The author of the commentary also pointed at other details that could pinpoint pre-menopausal (or younger patients in general) patients having increase risk for worse prognosis when compared to post-menopausal patients include: 1) younger women may be diagnosed late due to higher breast density; 2) younger women are more likely to have subtypes with worse prognosis such as HE negative, HER2 positive and TNBC; 3) older women are more likely to be diagnosed by screening due to lower breast density 4) older women are more likely to be diagnosed with HR-positive disease (which have more available therapeutic options that can decrease the risk of recurrence) [31].

There are certain limitations in this study, one problem is the sample size. Although there are analyses done in this paper that showed statistically significant results, the significance of the other subgroups could be confirmed by increasing the power of the study (increasing the population group). There was also paucity in patients with TNBC but with HER2 IHC samples of +1/+2/FISH negative, hence, meaningful statistical analysis cannot be done for this group. The access of ideal treatments and prognostic tests for breast cancer are also lacking in the study population. The majority of the patients in the Philippine General Hospital are indigent patients that have limited financial support, thereby limiting their access and compliance to certain expensive interventions such as gonadotropin receptor agonists, immunotherapy, Oncotype-DX testing and timely radiologic imaging.

A significant limitation of the current study stems from the substantial number of surveillance patients lost to follow-up. The recent COVID-19 pandemic has greatly impacted the ability to track these patients, making the update of their patient status a challenging task. This loss to follow-up has inevitably affected the comprehensiveness and potentially the accuracy of the study's findings.

In light of the findings seen in this study, the following recommendations can be made to bridge the knowledge gap in this field:

1. Create a separate study on Filipino patients with HER2 low status, since the results of this study do not reflect what is seen in scientific literature.
2. Create a larger study for locally advanced HER2 low patients.
3. Create studies with larger samples that will tackle the difference in survival of patients with HER2 +1 and HER2 +2/FISH negative patients.

## Conclusion

The 3-year DFS for the Luminal HER2 0 (negative) group is 84.4% while the Luminal HER2 +1 group showed a lower 3-year DFS at 81.8%. Most notably, the Luminal HER2 +2/FISH negative group demonstrated the lowest 3-year DFS at 52.9%. The study identified a significant lower 3-year DFS in HER2 +2/FISH negative patients in both early stage and locally advanced breast cancer. The findings underscore the importance of HER2 status in treatment planning and may inform future therapeutic strategies, including targeted therapies like T-Dxd.

## Conflicts of interest

The authors do not declare any conflicts of interest in this study.

## Funding

Personal funding.

## Figures and Tables

**Table 1.** Descriptive statistics.

	*N* (%)
**Population**	**N = 138**
Mean age (SD)	55.8 (10.4)
Range	26–74 years old
**Stage**	**N (%)**
I II III	10 (7.24%) 58 (42.03%) 70 (50.72%)
Early stage Locally-advanced	96 (69.57%) 42 (3.43%)
**HER2 status:**	
0 +1 +2/ FISH negative	94 (68.12%) 22 (15.94%) 22 (15.94%)
**Intrinsic subtype:**	
Luminal Basal type/TNBC	116 (84.06%) 22 (15.94%)
**Menopausal state**	
Pre-menopause Post-Menopause	59 (42.75%) 79 (57.24%)
**Treatment history (early-stage):**	
Neoadjuvant cytotoxic chemotherapy: Adjuvant cytotoxic chemotherapy: Adjuvant hormonal therapy*:	4 (4.17%) 80 (83.33%) 76 (96.20%)
**Treatment history (locally-advanced)**	
Neoadjuvant cytotoxic chemotherapy: Adjuvant cytotoxic chemotherapy: Adjuvant hormonal therapy*: Adjuvant CDK 4/6 therapy*:	24 (57.14%) 18 (42.86%) 37 (97.37%) 0 (0%)

SD, Standard Deviation; FISH, fluorescent in situ hybridisation; *Luminal group only

**Table 2.** Cox proportion analysis for age (with HER2 0 as reference).

	coef	exp(coef)	se(coef)	*z*	Pr(>|z|)
**HER2 +1**	−0.004567	0.995444	0.416758	−0.011	0.9913
HER2 +2/FISH negative	1.504724	**4.502909**	0.362862	4.147	3.37e-05 ***
Age	−0.03026	0.970194	0.015731	−1.923	0.0544

Concordance = 0.665 (se = 0.044 )

Likelihood ratio test= 16.58 on 3 df, p = 9e-04

Wald test = 19.44 on 3 df, p = 2e-04

Score (logrank) test = 21.95 on 3 df, p = 7e-05

*** p-value highly significant (less than 0.001)

**Table 3.** Cox proportion analysis for menopause (with HER2 0 as reference).

	coef	exp(coef)	se(coef)	*z*	Pr(>|z|)
**HER2 +1**	−0.1565	0.8552	0.4130	−0.379	0.70482
HER2 +2/FISH negative	1.3899	**4.0145**	0.3487	3.986	6.73e-05 ***
Menopause	−0.8239	0.4387	0.3067	−2.686	0.00723 **

Concordance = 0.675 (se = 0.038 )

Likelihood ratio test = 20.48 on 3 df, p = 1e-04

Wald test = 22.78 on 3 df, p = 4e-05

Score (logrank) test = 25.69 on 3 df, p = 1e-05

** p-value moderately significant (less than 0.01)

*** p-value highly significant (less than 0.001)

## Appendix

### Statistical results from RStudio

#### TNBC versus conventional HER2 negative

Group = TNBC

time n.risk n.event survival std.err lower 95% CI upper 95% CI

0 22 0 1.000 0.0000 1.000 1.000

6 22 0 1.000 0.0000 1.000 1.000

12 21 1 0.955 0.0444 0.871 1.000

18 20 1 0.909 0.0613 0.797 1.000

24 17 2 0.818 0.0822 0.672 0.996

30 14 1 0.767 0.0916 0.607 0.969

36 11 0 0.767 0.0916 0.607 0.969

Group= HER2 negative time n.risk n.event survival std.err lower 95% CI upper 95% CI

0 116 0 1.000 0.0000 1.000 1.000

6 112 5 0.957 0.0189 0.921 0.995

12 106 8 0.888 0.0293 0.832 0.947

18 100 4 0.853 0.0328 0.791 0.920

24 95 4 0.819 0.0358 0.752 0.892

30 90 3 0.792 0.0377 0.722 0.870

36 86 0 0.792 0.0377 0.722 0.870

#### TNBC versus HER2 0 versus HER2 low

Group=TNBC time n.risk n.event survival std.err lower 95% CI upper 95% CI

0 22 0 1.000 0.0000 1.000 1.000

6 22 0 1.000 0.0000 1.000 1.000

12 21 1 0.955 0.0444 0.871 1.000

18 20 1 0.909 0.0613 0.797 1.000

24 17 2 0.818 0.0822 0.672 0.996

30 14 1 0.767 0.0916 0.607 0.969

36 11 0 0.767 0.0916 0.607 0.969

Group=HER2 0 time n.risk n.event survival std.err lower 95% CI upper 95% CI

0 77 0 1.000 0.0000 1.000 1.000

6 77 0 1.000 0.0000 1.000 1.000

12 73 6 0.922 0.0305 0.864 0.984

18 69 2 0.896 0.0348 0.830 0.967

24 65 4 0.844 0.0414 0.766 0.929

30 63 0 0.844 0.0414 0.766 0.929

36 59 0 0.844 0.0414 0.766 0.929

Group=HER2 low time n.risk n.event survival std.err lower 95% CI upper 95% CI

0 39 0 1.000 0.0000 1.000 1.000

6 35 5 0.872 0.0535 0.773 0.983

12 33 2 0.821 0.0615 0.708 0.950

18 31 2 0.769 0.0675 0.648 0.914

24 30 0 0.769 0.0675 0.648 0.914

30 27 3 0.692 0.0739 0.562 0.853

36 27 0 0.692 0.0739 0.562 0.853

#### Luminal HER2 0, HER2 +1, HER2 +2/FISH negative

Group= Luminal HER2 0 time n.risk n.event survival std.err lower 95% CI upper 95% CI

0 77 0 1.000 0.0000 1.000 1.000

6 77 0 1.000 0.0000 1.000 1.000

12 73 6 0.922 0.0305 0.864 0.984

18 69 2 0.896 0.0348 0.830 0.967

24 65 4 0.844 0.0414 0.766 0.929

30 63 0 0.844 0.0414 0.766 0.929

36 59 0 0.844 0.0414 0.766 0.929

Group= Luminal HER2 +1 time n.risk n.event survival std.err lower 95% CI upper 95% CI

0 22 0 1.000 0.0000 1.000 1.000

6 21 1 0.955 0.0444 0.871 1.000

12 20 1 0.909 0.0613 0.797 1.000

18 19 1 0.864 0.0732 0.732 1.000

24 19 0 0.864 0.0732 0.732 1.000

30 18 1 0.818 0.0822 0.672 0.996

36 18 0 0.818 0.0822 0.672 0.996

Group= Luminal HER2 +2/FISH negative time n.risk n.event survival std.err lower 95% CI upper 95% CI

0 17 0 1.000 0.000 1.000 1.000

6 14 4 0.765 0.103 0.587 0.995

12 13 1 0.706 0.111 0.519 0.959

18 12 1 0.647 0.116 0.455 0.919

24 11 0 0.647 0.116 0.455 0.919

30 9 2 0.529 0.121 0.338 0.829

36 9 0 0.529 0.121 0.338 0.829

#### Early stage luminal HER2 0, HER2 +1 and HER2 +2/FISH negative

Group = Luminal HER2 0 time n.risk n.event survival std.err lower 95% CI upper 95% CI

0 54 0 1.000 0.0000 1.000 1.000

6 54 0 1.000 0.0000 1.000 1.000

12 52 4 0.926 0.0356 0.859 0.998

18 49 1 0.907 0.0394 0.833 0.988

24 46 2 0.870 0.0458 0.785 0.965

30 45 0 0.870 0.0458 0.785 0.965

36 41 0 0.870 0.0458 0.785 0.965

Group= Luminal HER2 +1 time n.risk n.event survival std.err lower 95% CI upper 95% CI

0 16 0 1.000 0.0000 1.000 1

6 16 0 1.000 0.0000 1.000 1

12 15 1 0.938 0.0605 0.826 1

18 14 1 0.875 0.0827 0.727 1

24 14 0 0.875 0.0827 0.727 1

30 13 1 0.812 0.0976 0.642 1

36 13 0 0.812 0.0976 0.642 1

Group= Luminal HER2 +2/FISH negative time n.risk n.event survival std.err lower 95% CI upper 95% CI

0 11 0 1.000 0.000 1.000 1.000

6 10 2 0.818 0.116 0.619 1.000

12 9 1 0.727 0.134 0.506 1.000

18 8 0 0.727 0.134 0.506 1.000

24 8 0 0.727 0.134 0.506 1.000

30 6 2 0.545 0.150 0.318 0.936

36 6 0 0.545 0.150 0.318 0.936

#### Locally advanced luminal HER2 0, HER2 +1 and HER2 +2/FISH negative

Group= Luminal HER2 0 time n.risk n.event survival std.err lower 95% CI upper 95% CI

0 23 0 1.000 0.0000 1.000 1.000

6 23 0 1.000 0.0000 1.000 1.000

12 21 2 0.913 0.0588 0.805 1.000

18 20 1 0.870 0.0702 0.742 1.000

24 19 2 0.783 0.0860 0.631 0.971

30 18 0 0.783 0.0860 0.631 0.971

36 18 0 0.783 0.0860 0.631 0.971

Group= Luminal HER2 +1 time n.risk n.event survival std.err lower 95% CI upper 95% CI

0 6 0 1.000 0.000 1.000 1

6 5 1 0.833 0.152 0.583 1

12 5 0 0.833 0.152 0.583 1

18 5 0 0.833 0.152 0.583 1

24 5 0 0.833 0.152 0.583 1

30 5 0 0.833 0.152 0.583 1

36 5 0 0.833 0.152 0.583 1

Group= Luminal HER2 +2/FISH negative time n.risk n.event survival std.err lower 95% CI upper 95% CI

0 6 0 1.000 0.000 1.000 1

6 4 2 0.667 0.192 0.379 1

12 4 0 0.667 0.192 0.379 1

18 4 1 0.500 0.204 0.225 1

24 3 0 0.500 0.204 0.225 1

30 3 0 0.500 0.204 0.225 1

36 3 0 0.500 0.204 0.225 1
